# The association between first trimester micronutrient intake, *MTHFR* genotypes, and global DNA methylation in pregnant women

**DOI:** 10.3109/14767058.2011.564242

**Published:** 2011-03-28

**Authors:** Michele La Merrill, Luisa Torres-Sánchez, Rubén Ruiz-Ramos, Lizbeth López-Carrillo, Mariano E Cebrián, Jia Chen

**Affiliations:** 1 Department of Preventive Medicine, Mount Sinai School of Medicine, New York, NY 10029, USA; 2 Instituto Nacional de Salud Pública, Av Universidad 655, Col Sta Maria Ahuacatitlan, Cuernavaca, Morelos, México CP 62100; 3 Departamento de Toxicología, CINVESTAV, México, Distrito Federal, México; 4 Department of Pediatrics, Mount Sinai School of Medicine, New York, New York 10029, USA

**Keywords:** Vitamin B6, epigenetics, one carbon metabolism, MTHFR 677, micronutrient deficiency

## Abstract

**Objective:**

Our aim was to evaluate possible associations between consumption of micronutrients involved in one-carbon metabolism, *MTHFR* genotypes, and global DNA methylation in pregnant women.

**Methods:**

A semi-quantitative dietary questionnaire was administered to 195 women during their first trimester in Morelos, Mexico. Two functional polymorphisms of the key folate-metabolizing gene, i.e. *MTHFR* 677 C>T and 1298 A>C, as well as global DNA methylation were assessed in peripheral blood drawn during the interview.

**Results:**

Independent of maternal age and caloric intake, vitamin B_6_ deficiency was associated with 1.8 fold increased risk of hypomethylation in women carrying the *MTHFR* 677 T allele.

**Conclusions:**

There exists a subpopulation that is more susceptible to B vitamin deficiencies.

## Introduction

Fetal growth places a high demand on the regulation of DNA synthesis and transcription. The regulation of gene transcription is partially dependent on DNA methylation, which utilizes methyl groups supplied by micronutrients in the one-carbon metabolic pathway. Consequently, inadequate consumption of methyl-related micronutrients, e.g. vitamins B_2_ (riboflavin), B_6_ (pyridoxine), B_9_ (folate), B_12_ (cobalamin), betaine, choline, and methionine, may result in aberrant DNA methylation. The possibility that aberrant DNA methylation is a mechanism in which micronutrient deficiencies lead to clinical manifestations remains relatively underexplored given the number of clinical manifestation that can result from deficiencies. Indeed, maternal deficiencies in some of these micronutrients contribute to neural tube defects (B_6_, B_9_, B_12_), cardiac defects (B_6_, B_9_), premature birth (B_9_), anemia (B_6_, B_9_, B_12_), teratogenesis (B_12_), neurological abnormalities such as neonatal demyelination (B_12_), convulsions (B_6_), brain atrophy (B_12_), cardiovascular disease (B_6_, B_9_), and cancer (B_6_, B_9_, B_12_) [1-3].

The rate of one-carbon metabolism is limited by methy-lenetetrahydrofolate reductase (MTHFR). MTHFR irreversibly catalyzes methyl donation of 5,10-methylene-tetrahydrofolate to form 5-methyl-tetrahydrofolate, which in turn provides a methyl group to homocysteine to form methionine [4]. Catalytic activity of MTHFR is reduced to 45% and 68% of the wild type activity when *MTHFR* is homozygous for the *MTHFR* variants 677T and 1298C, respectively [5]. Numerous evidence indicates that reduced MTHFR activity associated with these variants is associated with decreased DNA methylation [6]. For instance, *MTHFR* 677 TT carriers have reduced DNA methylation in their placentas [7]. We previously reported that low activity *MTHFR* 677 and 1298 variants increase the risk of spontaneous abortion [8]. Further, *MTHFR* variants are frequently reported to interact with micronutrients to influence the efficiency of this pathway. For instance, we have found that maternal folate deficiencies increased the risk of poor mental development only in offspring of mothers carrying the *MTHFR* 677 TT genotype [9]. Further, both *MTHFR* 677 C>T and 1298 A>C polymorphisms interact with methyl-donating micronutrient levels, e.g. B_2_, B_12_ and methionine, on risk of cancer [10,11].

Despite the numerous health consequences of vitamin B deficiencies, little research links these micronutrient deficiencies and poor health outcomes to DNA methylation. One recent study found that maternal deficiency in vitamin B and decreased DNA methylation (hypomethylation) in nervous tissue was significantly associated with neural tube defects in stillborns [12]. Unfortunately, this study did not examine whether *MTHFR* allele status modified these effects. To the best of our knowledge, the influence of one-carbon metabolism on DNA methylation in pregnant woman has never been studied. Here we seek to identify whether deficiencies in a comprehensive panel of one-carbon metabolism micronutrients and inherited genetic variability in *MTHFR* work together to influence the extent of global DNA methylation in pregnant women.

## Methods

### Population

A cohort of 996 women from the State of Morelos, in Mexico, was enrolled in 2001. These reproductive-age women were included if they fulfilled five criteria: (1) expressed intention to live in select counties from 2001 to 2003, (2) were not breast feeding, (3) did not use anticonvulsive drugs, (4) had no renal-, hepatic-, digestive- or thyroid-pathology history, and (5) did not use permanent contraceptive. Further cohort details are documented elsewhere [13]. Briefly, these women were enrolled in the cohort prior to conception. As part of their prenatal follow-up during a structured interview in the first trimester, maternal dietary information was collected and a blood sample was obtained. For the present study, we have complete information from 195 women. These women were slightly younger on average than the women of the original cohort (21.7 *vs*. 22.8 years old, respectively, *p*-value < 0.01). This study was approved by the Institutional Review Board of the National Institute of Public Health in Mexico.

### Diet

Maternal dietary habits during the first trimester of pregnancy were evaluated using a validated semi-quantitative food frequency questionnaire [14,15]. Briefly, the questionnaire included information about 95 foods divided into 11 groups (milk products, fruits, meats, vegetables, legumes, cereals, drinks, oils, local dishes, sodas, and candies) and it considered 10 categories of consumption frequency, from ‘never’ up to ‘six times a day’ [9,14,16]. Consumption of fruits and vegetables was adjusted according to availability in the market during the year. For example, in the case of plums, which were only available for 6 months of the year, the reported frequency was divided by two.

The dietary intake of B vitamins (B_2_, B_6_, B_9_, and B_12_) was estimated through the computer program developed by the University of Texas (Food Intake Analysis System 3.0) as previously described [14]. Specifically, liver, beans, lentils, green beans, peas, tomatoes, spinach, lettuce, potatoes, cauliflower, broccoli, chili peppers, beets, carrots, avocados, oranges, papayas, mangos, strawberries, bananas, tangerines, melons, plums, pears, pineapples, apples, peaches, grapes, lamb, tuna, pork, and steak were considered dietary sources of vitamins B_2_, B_6_, and B_9_. Further, the estimated dietary intake of vitamin B_12_ was based in the consumption of liver, sardines, tuna, pork, lamb, steak, fish, chocolate, beer, as well as red and white wines. For the purpose of this study, the nutrient tables were updated with betaine and choline contents reported by the US Department of Agriculture [17] and with methionine from the food composition tables of the ‘Salvador Zubirán’ National Institute of Nutrition [18].

### Genetic and epigenetic analyses

Maternal blood was collected for genetic and epigenetic analyses. Global DNA methylation (%) was ascertained by the LUminometric Methylation Assay method [19]. Briefly, blood DNA was co-digested with either HpaII and EcoRI or MspI and EcoRI (New England Biolabs) and DNA fragments were sequenced (GTGTCACATGTGTG) using a Q24 pyrosequencer (Qiagen) [20]. Lambda DNA and lambda treated with the CpG methyltransferase M.SssI, were used as negative and positive controls, respectively (New England Biolabs). Maternal *MTHFR* genotypes (C677T and A1298C) were determined by PCR-RFLP [9,16].

### Statistical analyses

The World Health Organization (WHO) recommended nutrient intake values for pregnant women were used to define the reference groups of the following nutrients: > 1.4 mg vitamin B_2_ (riboflavin), > 1.9 mg vitamin B_6_ (pyridoxine), > 600 *μ*g vitamin B_9_ (dietary folate equivalents), and > 2.6 *μ*g vitamin B_12_ (cobalamin) [21].

The WHO does not make recommendations for the intake of choline; therefore the reference group for choline was defined as daily intake that meets or exceeds 425 mg, based on the adequate intake recommended for women by the Institute of Medicine (IoM) [22]. Neither the WHO nor IoM has recommendations for the intake of betaine or methionine, and the reference groups for these micronutrients were defined as at or above the median daily value within the study population.

DNA methylation (%) was not normally distributed (Shapiro-Wilk *p*-value < 0.0001), and while squaring methylation resulted in a normal distribution, neither the DNA methylation (%) nor the transformed (%^2^) had homoscedasticity of variance, which violates linear regression assumptions. Therefore, DNA methylation was dichotomized where DNA methylation levels below the median DNA methylation level measured in this population were considered adverse outcomes, hereafter referred to as DNA hypomethylation [23]. The *MTHFR* 677 CC and 1298 AA genotypes were considered wild type because they are associated with maximal MTHFR catalytic activity [5]. *MTHFR* dominance was defined as the combination of the heterozygous- and the homozygous low catalytic activity-alleles compared to wild-type, e.g. *MTHFR* 677 TT and CT carriers compared to *MTHFR* 677 CC, and *MTHFR* 1298 CC and AC carriers compared to *MTHFR* 1298 AA carriers. Hardy-Weinberg equilibrium was assessed for *MTHFR* 1298 and *MTHFR* 677.

Relative risks (RR) of DNA hypomethylation associated with micronutrient deficiencies or *MTHFR* (dominance model of 677 T and 1298 C alleles) were estimated by multivariate general linear models [24]. As our *a priori* hypothesis was that micronutrient deficiencies and low activity *MTHFR* genotypes would increase the risk of DNA hypomethylation, no corrections were made for multiple comparisons [25,26]. Interaction between micronutrients and genotypes was only to be evaluated if there was a significant main effect of a micronutrient or *MTHFR* genotype on risk of DNA hypomethylation.

The following potential covariates were considered: maternal age (<20 years old, 20-24 years old, >24 years old), education (<12 years, > 12 years), parity (nulliparous, multi-parous), first trimester BMI (underweight, normal, overweight, obese), tobacco use during pregnancy (none, used), alcohol use during pregnancy (none, >1 cup/week), gestational age (estimated by reported date of last menstrual period), and offspring sex (obtained during the postpartum interview).

For each micronutrient or *MTHFR* model, we assessed potential confounding by three criteria. First we examined the χ^2^ test of independence (*p*-value < 0.1) between either the micronutrient or the genotype and potential confounders. Next, we evaluated the χ^2^ test of independence (*p*-value < 0.1) between DNA hypomethylation and potential confounders when the micronutrients status was restricted to adequate, or when genotypes were wild type. Finally, among potential confounders identified with χ^2^ test of independence (*p*-value < 0.1) in the first two steps, confounders were identified if they changed the *β* estimate of the micronutrient by greater than 10%. If a micronutrient significantly altered the RR of hypomethylation, we evaluated whether this association could be modified by *MTHFR* status. Multiplicative interaction of a micronutrient and *MTHFR* (dominance models of 677 T and 1298 C alleles) was assessed by the Breslow-Day test for homogeneity. Further evaluation of potential micronutrient effect modification by *MTHFR* status was assessed by stratifying the *MTHFR* dominance categories. Within each *MTHFR* stratum, the RR of hypomethylation due to a micronutrient independent of confounders was assessed [27]. All statistical analyses were performed using Statistical Analysis Software, Version 9 (Cary, NC).

## Results

The mean percent DNA methylation was 58.8% (standard deviation 11.4) and its median was 59.8 (range 10.8%-86.7%). The majority of women had less than 12 years of education, were nulliparous, and had a normal BMI in early pregnancy (81.0%, 84.6%, and 64.1%, respectively; Table I). Tobacco and alcohol use during pregnancy were reported by 10% and 16% of the women respectively (Table I).

**Table I:** Selected maternal characteristics of the population for which DNA methylation in maternal blood was evaluated (*n* = 195).

			DNA hypomethylation		
			
Maternal characteristics	*n*	%	*n*	Prevalence	Crude RR (95% CI)
Age					
<20 years old	64	32.8	35	54.7	1.00
20–24 years old	74	38.0	31	41.9	0.77 (0.54, 1.08)
≥24 years old	57	29.2	34	59.6	1.09 (0.80, 1.48)
Education					
≤12 years	158	81.0	81	51.3	1.00
>12 years	37	19.0	19	51.4	1.00 (0.71, 1.42)
Parity					
No previous pregnancy	165	84.6	87	52.7	1.00
≥1 previous pregnancy	30	15.4	13	43.3	0.82 (0.53, 1.27)
BMI					
Underweight	15	7.7	8	53.3	1.04 (0.63, 1.72)
Normal weight	125	64.1	64	51.2	1.00
Overweight	42	21.5	23	54.8	1.06 (0.77, 1.48)
Obese	13	6.7	5	38.5	0.75 (0.37, 1.52)
Tobacco use during pregnancy					
None	175	89.7	91	52.0	1.00
Used	20	10.3	9	45.0	0.86 (0.52, 1.43)
Alcohol use during pregnancy					
None	164	84.1	86	52.4	1.00
≥1 cup/week	31	15.9	14	45.2	0.86 (0.57, 1.30)
MTHFR 1298					
AA	158	81.0	78	49.4	1.00
AC + CC	37	19.0	22	59.5	1.20 (0.88, 1.64)
MTHFR 677					
CC	31	15.9	16	51.6	1.00
CT + TT	164	84.1	84	51.2	0.99 (0.68, 1.44)

*MTHFR* 677 C>T and 1298 A>C were in Hardy-Weinberg equilibrium (*p*-value > 0.4). *MTHFR* 677 CC, CT, and TT genotypes occurred in 16%, 51%, and 33% of the pregnant women, respectively. The crude risk of hypomethylation associated with *MTHFR* 677 CT and TT genotypes relative to the *MTHFR* 677 CC genotype was not significant (1.05; 95% CI: 0.71, 1.55 and 0.91; 95% CI: 0.60, 1.39, respectively). The prevalence of *MTHFR* 1298 AA and AC frequencies was 78% and 22%, respectively, with no observed *MTHFR* 1298 CC carriers. None of the maternal characteristics described in Table I were significantly associated with DNA hypomethylation risk.

Among the study population, 2.0 mg vitamin B_1_ 2.0 mg vitamin B_2_, 1.9 mg vitamin B_6_, 350.3 mg vitamin B_9_, 3.6 mg vitamin B_12_, 37.6 mg betaine, 333.4 mg choline, and 1,813.0 mg methionine were consumed on average daily. According to WHO and IoM guidelines, vitamin B_6_, choline, and vitamin B_9_ were the micronutrients in deficiency among the majority of pregnant women observed (61.0%, 78.0%, and 91.3%, respectively; Table II). Independent of maternal age and total caloric consumption, the inadequate intake of vitamin B_6_ was associated with a significant increase of nearly doubled risk of DNA hypomethylation (1.80; 95% CI: 1.24, 2.61; Table II). Deficiencies in the consumption of other micronutrients involved in one-carbon metabolism were not associated with risk of DNA hypomethylation in pregnant women (Table II).

**Table II:** Relative risks of DNA hypomethylation in maternal blood due to low levels of micronutrients in a Mexican pregnancy cohort (*n* = 195).

	Methylation		Risk Ratio (95% CI)	
		
Micronutrients (mg/day)	Hyper *n*	Hypo *n*	Unadjusted	Adjusted*
B_1_ (Thiamine)				
≥1.4	81	80	1.00	1.00
<1.4	14	20	1.18 (0.86, 1.63)	1.12 (0.75, 1.67)
B_2_ (Riboflavin)				
≥1.4	81	83	1.00	1.00
<1.4	14	17	1.08 (0.76, 1.54)	0.97 (0.63, 1.49)
B_6_ (Pyridoxine)				
≥1.9	46	30	1.00	1.00
<1.9	49	70	1.45 (1.06, 1.99)	1.80 (1.24, 2.61)
B_9_ (Folate)				
≥0.6	9	8	1.00	1.00
<0.6	86	92	1.10 (0.65, 1.85)	0.98 (0.56, 1.73)
B_12_ (Cobalamin)				
≥0.0026	62	68	1.00	1.00
<0.0026	33	32	0.94 (0.70, 1.27)	0.89 (0.65, 1.21)
Betaine				
≥31.108	52	46	1.00	1.00
<31.108	43	54	1.19 (0.90, 1.56)	1.15 (0.843, 1.56)
Choline				
≥425	23	20	1.00	1.00
<425	72	80	1.13 (0.79, 1.61)	1.01 (0.66, 1.53)
Methionine				
≥1755.311	51	46	1.00	1.00
<1755.311	44	54	1.16 (0.88, 1.53)	1.08 (0.74, 1.59)

* Adjusted by maternal age and total caloric consumption.

Vitamin B_6_ deficiency and *MTHFR* 677 (dominance model) significantly interacted on the risk of DNA hypomethylation during pregnancy (Breslow-Day test of homogeneity *p*-value *—* 0.02; Table III). Among *MTHFR* 677 TT and CT carriers, vitamin B_6_ deficiency increased the risk of DNA hypomethylation 1.79 times over the risk of hypomethylation associated with vitamin B_6_ adequacy (95% CI: 1.13, 2.85; Table III). However, vitamin B_6_ deficiency appeared to have little effect on DNA hypomethylation status compared to vitamin B6 adequacy in carriers of the CC genotype (wild-type) of *MTHFR* 677 (RR= 1.51, 95% CI: 0.57, 4.03; Table III). There was no significant interaction between *MTHFR* 1298 (dominance model) and vitamin B_6_ deficiency on risk of DNA hypomethylation (Breslow-Day *p*-value = 0.79; Table III).

**Table III:** Relative risks of DNA hypomethylation in maternal blood due to low levels of vitamin B_6_ in a Mexican pregnancy cohort stratified by MTHFR status(*N* = 195).

	MTHFR 677*†				MTHFR 1298*‡			
		
	CC		CT/TT		AA		AC/CC	
				
VitaminB_6_ (mg/d)	*n*	aRR	*n*	aRR	*n*	aRR	*n*	aRR
≥1.9	15	1.0	61	1.0	63	1.0	13	1.0
<1.9	16	1.51 (0.57, 4.03)	103	1.79 (1.13, 2. 85)	95	1.62 (1.01, 2.58)	24	2.24 (0.88, 5.67)

* Adjusted by maternal age and total caloric consumption.

† Breslow-Day test of homogeneity *p*-value= 0.02.

‡ Breslow-Day test of homogeneity *p*-value=0.79.

## Discussion

The prevalence of vitamin B_6_-, folate-, and choline-deficiencies was high among pregnant Mexican women of this study. One-carbon metabolism micronutrient deficiencies during pregnancy can have a negative impact on fetal development, such as increasing risk of neural tube defects and preeclampsia [1,2,28]. Perhaps these micronutrient deficiencies decrease the production and maintenance of proper DNA methylation patterns that critically support maternal health and fetal growth. Vitamin B_6_ deficiency was associated with a modest increase in risk of DNA hypomethylation in this study and elsewhere [29]. Although clinically defined vitamin B_6_ deficiency does not always lead to adverse clinical outcomes, our data suggest that vitamin B_6_ deficiency has a subclinical presentation, DNA hypomethylation.

Some pregnant women may have greater susceptibility to the consequences of micronutrient deficiencies due to their inherited genetic variabilities. In particular, our results indicate that the decreased MTHFR catalytic activity associated with *MTHFR* 677 T alleles may increase susceptibility to the effect of vitamin B_6_ deficiency on DNA hypomethylation in pregnant women. Decreased catalytic activity of MTHFR 1298 AC and CC may also increase the risk of DNA hypomethylation due to vitamin B_6_ deficiency; however, the combination of the low prevalence of wild type *MTHFR* 1298 carriers in this population along with the relatively weak biological effects of MTHFR 1298 AC and CC likely underpowered our ability to evaluate this [5].

Increased DNA hypomethylation in pregnant women who are vitamin B_6_ deficient and carriers of low activity *MTHFR* alleles is biologically plausible given that vitamin B_6_ is a necessary cofactor of serine hydroxymethyltransferase, which produces 5,10-methylenetetrahydrofolate, the MTHFR substrate that provides methyl groups for DNA methylation [30,31]. Other research suggests that the *MTHFR* 677 T allele and vitamin B_6_ deficiency interact to cause adverse health outcomes. Among carriers of the *MTHFR* 677 TT genotype, those with vitamin B_6_ deficiency had greater risk of cancers of the lungs and colon [10,32] as well as higher levels of plasma homocysteine [33,34], the later of which is associated with poor pregnancy outcomes [2,28]. The apparent genetic susceptibility to the consequences of vitamin B_6_ deficiency seen on risk of DNA hypomethylation here may further explain why not all vitamin B_6_ deficient individuals present with adverse health.

In what we believe to be the first study to examine the relationships between micronutrient intake, *MTHFR* status, and global DNA methylation in pregnant women, our results are consistent with the hypothesis that deficiencies in dietary vitamin B_6_ increase DNA hypomethylation more so in carriers of low activity genetic variants of *MTHFR* than in carriers of wild type MTHFR variants. The modest magnitude of DNA hypomethylation risk reported needs to be confirmed in larger prospective epidemiology studies to determine whether DNA methylation mediates the relationship between vitamin B_6_ deficiency, *MTHFR,* and adverse maternofetal health. Moreover, determination of the effect of vitamin B_6_ deficiency on the DNA methylation status of cells from MTHFR variant carriers would be particularly insightful.
